# Phenotypic and Genotypic Diversity of the Tomato Germplasm From the Lazio Region in Central Italy, With a Focus on Landrace Distinctiveness

**DOI:** 10.3389/fpls.2022.931233

**Published:** 2022-07-22

**Authors:** Barbara Farinon, Maurizio E. Picarella, Francesca Siligato, Roberto Rea, Paola Taviani, Andrea Mazzucato

**Affiliations:** ^1^Laboratory of Biotechnologies of Vegetable Crops, Department of Agriculture and Forest Sciences, University of Tuscia, Viterbo, Italy; ^2^ARSIAL, Regional Agency for the Development and the Innovation of Lazio Agriculture, Rome, Italy

**Keywords:** distinctiveness, flattened-ribbed fruits, regional landraces, tomato, SNP polymorphisms

## Abstract

Italy is a recognized secondary center of diversification for cultivated tomato (*Solanum lycopersicum* L.). The study of phenotypic and genetic diversity in landrace collections is important for germplasm conservation and valorization. Here, we set up to study the tomato germplasm collected in the region of Lazio in Central Italy, with a focus on the distinctiveness among landraces and the attribution of membership to unnamed accessions. Our regional collection included 32 accessions belonging to eight different locally recognized landraces and 19 unnamed accessions. All accessions were gathered from local farmers and are preserved in the collection held at the Regional Agency for the Development and the Innovation of Lazio Agriculture (ARSIAL) and at the University of Tuscia. We included 13 control genotypes comprising nine landraces from neighbor regions and four reference cultivars. The collection showed wide phenotypic variability for several qualitative and quantitative traits, such as leaf border and shape, inflorescence type, fruit shape, green shoulder, fruit weight (range 14–277 g), locule number (2–12), shape index (0.54–2.65), yield (0.24–3.08 kg/plant), and soluble solids (3.4–7.5°B). A few landraces showed uncommon phenotypes, such as potato leaf, colorless fruit epidermis, or delayed ripening. Multivariate analysis of 25 cardinal phenotypic variables separated the accessions into two distinct groups; accessions showing a flattened-ribbed fruit were distinguished from those with round to elongate fruits with smooth structure. Genotyping analysis of 7,720 SNPs was performed using the tomato array platform SolCAP, to point out the genetic relationship among the studied accessions. A neighbor-joining tree analysis allowed to confirm or deny phenotypic data and to assign some of the unnamed accessions to recognized groups. Allelic status at marker loci linked to resistance genes commonly used in breeding identified accessions putatively derived from modern material or commercial hybrids, thus not classifiable as landraces. Overall, this study provided the information useful to preserve, valorize, and juridically protect tomato local landraces from the Lazio region and will in addition be helpful to their improvement by breeding.

## Introduction

Cultivated tomato (*Solanum lycopersicum* L.) was introduced in Europe from Latin America at the beginning of the XVI century. It was only in the XVIII century that the species became popular for human consumption; such diffusion started first in Europe and then spread to North America and Asia (Mehta, 2017; OECD, 2017). In Italy, by virtue of the highly different pedoclimatic, cultural, and politic environments, the tomato found a secondary center of diversification and the empirical selection by farmers drove the development of a great variety of landraces differentiated for fruit shape, size, and end use of the fruit (Siviero, 2001; Mazzucato et al., 2008; García-Martínez et al., 2013). In the second half of the last century, the massive introduction of professionally improved varieties, first based on true breeding lines and later F_1_ hybrids, eliminated these traditional types from cultivation, and relegated them to the interest of local markets and home-consumption gardeners. The types of greater success, such as Cuore di Bue, San Marzano, and Costoluto fiorentino, spread over the whole national cultivation and have been massively grown till the mid of the XX century; such varieties are still nowadays listed in the National Plant Variety Register^1^ (last accessed on 20 April 2022).

In Italy, tomatoes with big flattened-ribbed fruits were mainly successful in the North (e.g., “Nostrano,” “Genovese,” “Riccio di Parma,” “Ladino di Pannocchia”) in contrast to those with small-mid size, elongate, elliptic, or round that found diffusion, especially in the South of the country (Soressi, 1969). In contrast to Northern Europe or America, where round-smooth tomatoes were preferred (Oltman et al., 2014), in Italy as in other Mediterranean countries, flattened-ribbed types found a wide diffusion. It seems that these tomatoes were the original introductions from Latin America (van Andel et al., 2022 and references therein). Notwithstanding, landraces have few chances to outstand modern hybrids and be adopted in intensive vegetable productions for large market targets, and it is acknowledged that they represent an important genetic material to be preserved and valorized, to satisfy the needing and perspective of niche agriculture and markets, that appreciate traditional products for their quality (Baldina et al., 2016; Casañas et al., 2017). In addition, landraces may be used as the gene donors for breeding novel varieties for the forthcoming needs, which included those imposed by climatic changes (Farinon et al., 2022).

A great number of studies addressed the estimation of the phenotypic and genetic variabilities of tomato collections, spanning from the wild to the feral and cultivated germplasm. Some of these were aimed to describe generalist collections, generally focused on phylogenetic assessments (Sim et al., 2012; Lin et al., 2014; Blanca et al., 2022a), or to establish gene-trait association by genome-wide association studies (GWAS) (Ruggieri et al., 2014; Zhao et al., 2019; Mata-Nicolás et al., 2020; Rodriguez et al., 2020; Gonzalo et al., 2021; Tripodi et al., 2021). Other studies were set up to describe more specific regional, national (Rao et al., 2006; Mazzucato et al., 2008; García-Martínez et al., 2013; Corrado et al., 2014; Sacco et al., 2015; Lázaro, 2018; Schouten et al., 2019; Castellana et al., 2020; Grozeva et al., 2020; Tamburino et al., 2020; Athinodorou et al., 2021), or international (Aflitos et al., 2014; Roohanitaziani et al., 2020; Blanca et al., 2022b) collections. However, a limited part of these studies was devoted to establishing and proposing the criteria for landrace distinctiveness and management, including assigning unnamed accessions to recognized typologies, discovering “false” landraces, or distinguishing the occurrence of homonymy or synonymy. Works aimed at assessing distinctiveness were usually addressed to single genotypes of interest (Mazzucato et al., 2010; Renna et al., 2019; Amado Cattáneo et al., 2020; Caramante et al., 2021; Aiese et al., 2022). Scant attention has also been devoted to separating recognized landraces from materials of uncertain identity or origin. When traditional tomato germplasm is collected in a defined area, researchers are usually meeting landraces that are recognized by the collective memory, that have a name, a tradition and are listed in official or volunteer repositories and catalogs. The collection of this material, which we therefore refer to as “named landraces,” is usually paralleled by that of genotypes that are claimed to be autochthonous and traditional by single farmer but are not supported by a collective cultivation history. Such materials, which we will refer to as “unnamed landraces,” may effectively be genuine autochthonous genotypes, whose collective memory is lost and the seed maintenance confined to a single farmer, or can often be recent introductions or selections that are *bona fide* claimed as landraces. Here, we set up to study the phenotypic and molecular variabilities of tomato landraces autochthonous of the specific region of Lazio in Central Italy to describe the existing diversity, to establish distinctiveness criteria among named landraces and to draw proposition of inclusion or rejection for material which is not accompanied by a collective memory of usage.

## Materials and Methods

### Plant Material

Morphological and molecular genetic diversities were assessed in 64 accessions of cultivated tomato (*Solanum lycopersicum* L.), including 51 landraces from Lazio in Central Italy, nine from neighbor regions, and four cultivars used as references (Supplementary Table 1). All accessions were present in the collection held at the Regional Agency for the Development and the Innovation of Lazio Agriculture (ARSIAL) and at the Department of Agriculture and Forest Sciences of the University of Tuscia. Accession V710292 (NAG) was originally obtained from the C. M. Rick Tomato Genetics Resource Center (TGRC), University of California, Davis, United States (LA2661). The Italian landraces were either gathered from the farmers or from local seed markets.

Plants were grown at two locations in the Lazio region: the Experimental Farm of the University of Tuscia at Viterbo, Lazio, Italy (42°25′07″ N, 12°06′34″ E, 326 m a.s.l.) and the ARSIAL Experimental farm at Alvito (FR), Lazio, Italy (41°41′24″ N, 13°44′52″ E, 482 m a.s.l.), in the same growing season (2021). A total of four plants per accession were arranged in twin rows and grown with standard agronomic practices for fresh market tomatoes. All genotypes were grown on tutors with a single shoot, lateral shoots were weekly removed, and plants were left to open pollination.

Named varieties were identified through three different official sources. The Voluntary Regional Register issued by the regional Law 1/03/2000 – N.15 “Tutela delle risorse genetiche autoctone di interesse agrario^2^” (last accessed on 21 January 2022) listed the landraces “Scatolone di Bolsena,” “Spagnoletta di Gaeta e Formia,” and “Da secca di Minturno.” The national list of “Traditional agro-food products” (PAT^3^, last accessed on 21 January 2022) listed in addition “Corno di toro,” “Fiaschetta di Fondi,” “Ovalone di Rieti,” and “Perino di Sperlonga.” No product from Lazio was found among the Slow Food Presidia^4^ (last accessed on 21 January 2022), whereas the landrace “Pantano romanesco” is still included in the National Plant Variety Register (see text footnote 1) (last accessed on 20 April 20, 2022).

### Phenotypic Data

At the Viterbo field, 40 qualitative and quantitative morpho-physiological descriptors were scored or calculated as detailed in Supplementary Table 2. At the vegetative level, on a single plant basis data on growth habit (GH, score), height of the first (H1, cm) and second (H2) nodes and of the total plant (HT) measured 60 days after transplanting (DAT), leaf shape (LS, score), leaf border (LB, score), and leaf attitude (LA, score) were detected. The flowering (FD) and ripening (RD) date were recorded as the DAT to the first open flower and to the first ripe fruit, respectively. At 60 DAT, the inflorescence type (IN) and determinacy (LI), the stigma position (SP), and the presence of abscission zone in the flower pedicel (JT) were scored. At the ripening of the second truss, the following fruit traits were scored; green shoulder (GS), fruit load (FL), external fruit color (FC), fruit shape (FS), fruit skin color (SC), fruit shoulder shape (SH), ribbing at calix end (RI), shape of scar (SS), shape of stylar pole (SY), easiness to detach (EA), occurrence of blossom-end rot (BR), and of radial (RC) and concentric (CC) cracking. A total of eight representative ripe fruits were harvested for each accession to individually measure fruit weight (FW, g), fruit polar (PD, mm) and equatorial (ED, mm) diameter, locule number (LN), pericarp thickness (PE, mm), and occurrence of puffiness (PA, score). The firmness (FF) of each fruit was estimated with a triplicate measure with a handheld Fruit Hardness Tester (53215, Turoni, Forlì, Italy). The juice obtained to extract the seeds was used for measuring soluble solid content (°B, degree Brix) by a digital refractometer (MA871, Milwaukee, Milwaukee Instruments, Inc., NC, United States). For each accession, the total number of fruits (NF) was counted on the four evaluated plants. Additionally, the following variables were calculated: internode length [IL, (H2 – H1)], flowering-ripening interval [RF, (RD – FD)], fruit shape index [SI, (PD/PE)], pericarp thickness index [PI, (PE/(PD + ED)/2))], and total yield [TY, (NF × FW)].

At the Alvito field, only FW, PD, ED, LN, and PE were measured; SI and PI were thereafter calculated as detailed above.

### Genotypic Data

For each accession, genomic DNA was extracted from 35 mg of frozen leaves with a modified protocol of the cetyltriethylammoniumbromide (CTAB) extraction method (Doyle and Doyle, 1990). DNA quantity and quality were evaluated by a Multiskan SkyHigh Microplate Spectrophotometer (Thermo Fisher Scientific, Waltham, MA, United States) at 260/280 and 260/230 OD ratios. Genotyping was carried out at VHL Genetics (Wageningen, Netherlands^5^) using 150 ng of freeze-dried DNA per accession and the tomato array platform SolCAP developed in the framework of the Solanaceae Coordinated Agricultural Project from NIFA/USDA and based on the ILLUMINA Infinium^®^ array Technology (Sim et al., 2012). The Illumina assay and subsequent SNP calling were performed as previously described (Ruggieri et al., 2014).

### Analysis of Phenotypic Data

To evaluate the morpho-physiological descriptors and detect correlations among them, a principal component analysis (PCA) was performed using the factoextra package implemented in R version 4.1.1^6^. For this, 25 scale and ordinal variables measured at the Viterbo field were considered, whereas variables used to determine calculated variables as well as nominal qualitative variables were excluded from the analyses. The set of accessions was split into the “flat” and “non-flat” type groups. A PCA based on the separate groups was also carried out. The distribution of nominal qualitative traits was studied separately for the two morphological groups.

For eight quantitative variables (i.e., IL, FD, FW, SI, LN, PI, FI, and °B), the distribution across the whole population was reported and the differences among named varieties with at least two accessions estimated through the analysis of variance adopting the general linear model (GLM) and Duncan’s multiple range test for mean separation. To meet, or approach, the homoscedasticity requirement, variables IL and SI were subjected to logarithmic transformation, and FW in square root. Parametric analyses were conducted with transformed variables, although means were reported in the original measurement units. All statistics were performed by SAS (SAS Institute, 2015). The Spearman’s rank correlation coefficients among variables were calculated based on the mean values of the Viterbo field.

For the variables such as FW, PD, ED, SI, LN, and PI scored in the two fields, the occurrence of Genotype × Environment (G × E) interaction was estimated by two-way ANOVA and linear regression analysis. For the same variables, and separately for the two fields, broad-sense heritability (h^2^_B_) was calculated as σ^2^_gen_/σ^2^_tot_ using variance estimates derived from one-way ANOVA. σ^2^_gen_ was calculated as (σ^2^_between_-σ^2^_within_)/k where k was the number of replicates for each observation. σ^2^_within_ was considered an estimate of σ^2^_e_. σ^2^_tot_ was σ^2^_gen_ + σ^2^_e_.

### Analysis of Genotypic Data

Single-nucleotide polymorphism data obtained in diploid mode were used to evaluate the genetic relationships among the studied accessions. First, markers with more than 10% missing genotypes, minor allele frequency (MAF) < 0.02, and observed heterozygosity higher than 25% were discarded. Moreover, taxa with more than 20% missing genotypes and observed heterozygosity higher than 25% were also filtered out. Heterozygosity was calculated and a neighbor-joining tree generated using TASSEL ver. 5.0 (Bradbury et al., 2007). The allelic status at marker loci linked to commonly used resistance genes (Sim et al., 2012) was manually screened.

STRUCTURE version 2.3.4 (Pritchard et al., 2000) was used to delineate the clusters of individuals based on their genotypes at multiple loci, adopting the “admixture model,” a burn-in period of 5,000 iterations, and a Bayesian Markov Chain Monte Carlo (MCMC) chain run for 50,000 steps. The SolCAP SNP dataset was pruned to a limited number of unlinked loci using a selection of 384 markers (Gill et al., 2019). The number of clusters (k) was selected using Structure Harvester (Earl and vonHoldt, 2012).

To evaluate the present collection in a broader genetic dataset, we run the neighbor-joining analysis with the previously described parameters after adding 40 additional genotypes from a published work (Sacco et al., 2015), including Italian, European, and Latin American landraces, reference cultivars, and breeding lines (Supplementary Table 3).

## Results

### Multivariate Analysis of the Whole Collection

A total of ten qualitative and 15 quantitative descriptors of vegetative and reproductive phenotypes (Supplementary Table 2) were processed through PCA to evaluate the overall variability across the 64 analyzed accessions (Supplementary Table 1). The first six components of the PCA explained about 68% of the total variance (Supplementary Figure 1A). The first two components covered about 40% of the total variance, with the first accounting for almost 25% and the second for 14.8% (Supplementary Figure 1A). According to the variable contribution (Supplementary Figure 1B and Supplementary Table 4), ED, LN, and PD were the main factors discriminating the analyzed accessions; indeed, ED and LN accounted for 14% of the total variation in the first component, and PD accounted for 16% of the total variation in the second component.

**FIGURE 1:**
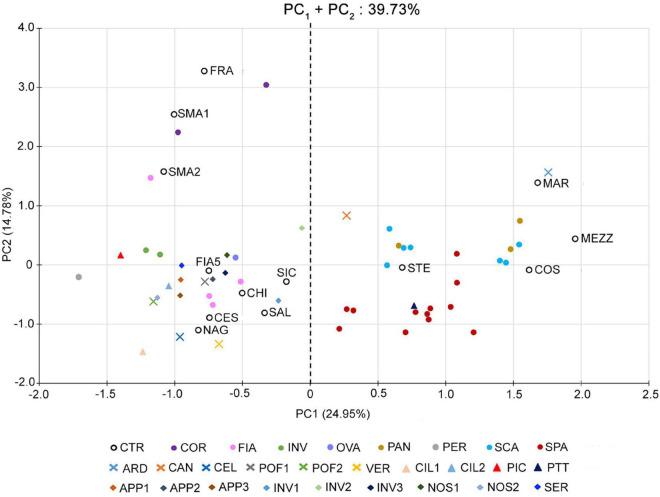
Multivariate analysis of the phenotypic variability of the 64 analyzed tomato genotypes. Scatter plot of the first (PC1) and second (PC2) principal components showing the variation for 25 vegetative and reproductive traits. Accessions are represented by differently colored symbols, as indicated in the legend. Accessions’ abbreviations are listed in Supplementary Table 1. The black dotted line at the zero divides the flat-type accessions (on the **right**) from the non-flat ones (on the **left**).

Such a PCA highlighted a clear separation of the accessions into two distinct groups along the first component (PC1) (Figure 1). The first group was localized in the positive dials of the PC1 axis and comprised 29 out of 64 accessions of the collection, including those of the named landraces SCA, SPA, and PAN, the control varieties STE, MAR, MEZ, and COS, and the unnamed accessions CAN, ARD, and PTT. According to the phenotypic data (Supplementary Table 5), these varieties are all characterized by fruits with a flat shape and a strong ribbing at the calyx end; therefore, they have been referred to as “flat” types.

The second group was placed in the negative dials of the PC1 axis, comprising the named landraces COR, FIA MIN, OVA, and PER, the control varieties CES, CHI, FIA5, FRA, NAG, SAL, *SIC*, and SMA, and 18 unnamed accessions (Figure 1). The accessions belonging to this group were referred to as “non-flat” types because of their fruit shape different from the first group (Supplementary Table 5).

### Diversity for Qualitative Traits

**FIGURE 2:**
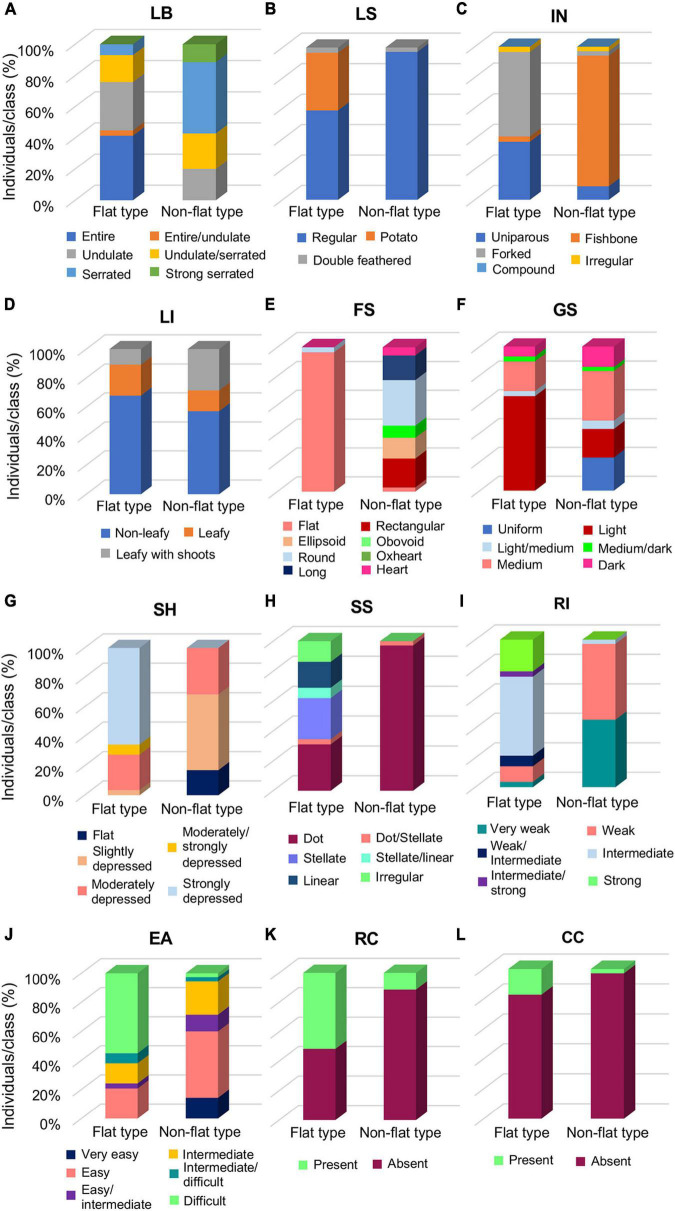
Distribution of selected qualitative descriptors between the flat- and the non-flat-type groups of the 64 analyzed tomato accessions. **(A)** Leaf border (LB), **(B)** leaf shape (LS), **(C)** inflorescence type (IN), **(D)** leafiness (LI), **(E)** fruit shape (FS), **(F)** green shoulder (GS), **(G)** fruit shoulder shape (SH), **(H)** shape of scar (SS), **(I)** ribbing at calix end (RI), **(J)** easiness to detach (EA), **(K)** radial cracking (RC), and **(L)** concentric cracking (CC). Each histogram reports the percentage of individuals falling in the respective class for the considered trait.

To evaluate the morphological differences distinguishing the flat and non-flat types, vegetative and fruit qualitative descriptors were separately evaluated for each group (Supplementary Table 5). Selected qualitative traits which appeared more divergent between the two groups, hence, more interesting for the group’s distinctiveness, are reported in Figure 2.

Among the vegetative traits, LB, LS, and IN (Figures 2A–C) were the most variable descriptors, with the flat group marked out by undulate or entire LB, and forked or uniparous IN, whereas in the non-flat-type group, serrated LB and fishbone IN were predominant (Figures 2A,C). Accessions with potato leaf were found only in the flat group (Figure 2B), mainly in the accessions of the SPA landrace, as well as in the unnamed accession PTT (Supplementary Table 5). For the most part, non-leafy inflorescence was observed in both groups, though a slightly higher percentage of leafy with shoots accessions were found in the non-flat-type than in the flat-type group (Figure 2D).

For fruit traits, as expected, the flat-type group included accessions almost entirely producing fruits with a flat shape, whereas greater variability of this trait was observed in the non-flat-type group, with a predominance for the round shape (Figure 2E). Furthermore, flat tomatoes predominantly showed a light GS (Figure 2F), a strongly depressed SH (Figure 2G), and a broad range of variability for the SS, the most represented of which were the stellate and dot, but linear and irregular scars were also found in some accessions of this group. On the contrary, the non-flat typologies were mainly characterized by medium GS, moderately or slightly depressed SH, and dot SS (Figures 2F–H). Predictably, the flat-type group produced fruits with an intermediate or strong RI, differently from the non-flat-type group’s fruits, characterized to have a weak or very weak RI (Figure 2I). An opposite trend between the two groups was observed for EA, which resulted more difficult for the flat-type cluster and easier for the non-flat-type cluster (Figure 2J). Noteworthy, the flat-type group showed a higher level of RC than the non-flat-type one (Figure 2K); a similar trend was also found for the CC, although it was generally less evident among all analyzed varieties of the collection (Figure 2L).

All the studied accessions showed fruits with red FC except CAN that presented the *colorless fruit epidermis* (*y*) variation (pink FC) and INV2, INV3, and SER that presented delayed ripening phenotypes (green or orange FC; Supplementary Table 5).

### Diversity for Quantitative Traits

**FIGURE 3:**
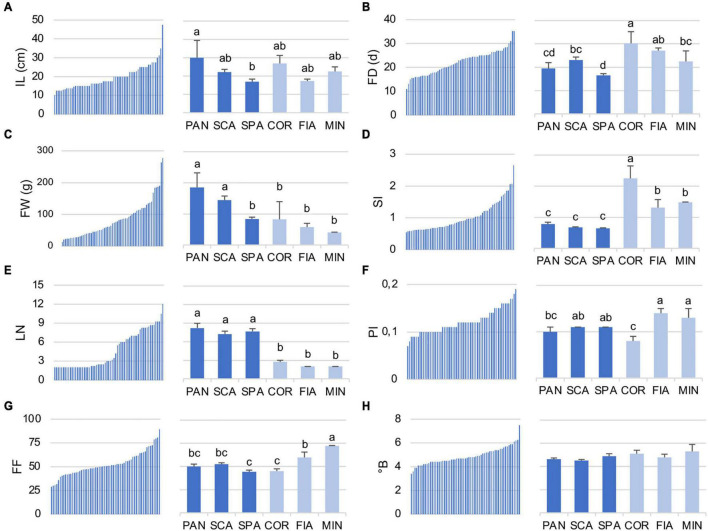
Distribution of selected quantitative traits in the 64 analyzed tomato accessions **(A)** internode length (IL), **(B)** flowering date (FD), **(C)** fruit weight (FW), **(D)** shape index (SI), **(E)** locule number (LN), **(F)** pericarp index (PI), **(G)** firmness (FI), and **(H)** total soluble solids (°B). For each descriptor, the range of values within the entire collection (left) and the ANOVA among the named landraces (right) are reported. Flat and non-flat named landraces are represented in dark and light blue, respectively. PAN, Pantano; SCA, Scatolone; SPA, Spagnoletta; COR, Corno di toro; FIA, Fiaschetta; MIN, Da secca di Minturno. In the right subpanels, means indicated by different lowercase letters are significantly different (*p* ≤ 0.05) after Duncan’s multiple range test.

Regarding the quantitative traits, the diversity for IL, FD, FW, SI, LN, PI, FI, and°B is reported in detail (Figure 3 and Supplementary Table 6). IL value ranged from 10.0 to 47.5 cm among the accessions of the entire collection. SPA7 was the accession with the lowest IL, whereas PAN2 was that with the highest value (Supplementary Table 6). Accordingly, among the named landraces, IL was significantly higher for PAN genotype, whereas SPA had the lowest value in comparison with the other named landraces of the collection (Figure 3A). In contrast, for FD, a marked difference between the two groups was detected, being the named flat types earlier in flowering than the non-flat ones. Particularly, SPA landraces resulted in those with the significantly lower FD, whereas the COR accessions were the latest to flower (Figure 3B). When the entire collection was considered, FD ranged from a minimum of 11.0 DAT (SPA1) to a maximum of 35.3 DAT, observed in the control genotype FRA as well as in the named landrace COR1 (Figure 3B and Supplementary Table 6).

The highest differences between the flat-type and non-flat-type groups were though related to fruit descriptors. With respect to dimension, flat-type accessions presented higher FW (range for the entire collection, 276.5–13.6 g) in comparison with the non-flat-type ones. Indeed, the biggest fruit of the studied population was found for the unnamed accession ARD, included in this group. Among the named flat-type landraces, SPA showed an FW value significantly lower on average, than PAN and SCA, and more similar to the non-flat-type group (Figure 3C). As expected, the most divergent fruit trait was SI, which was significantly lower for all three named flat landraces PAN, SCA, and SPA than the non-flat varieties (Figure 3D). The accession with the highest SI value (2.65) was COR2, whereas SPA3 was the one with the lowest (0.54). In parallel, the flat-type group had an LN significantly higher than the non-flat-type cluster (Figure 3E); in fact, the highest LN values of the population were found for the unnamed accession ARD (12.0) and for the named landrace SPA9 (10.5), whereas the non-flat-type group showed a trend with a higher PI (range for the entire collection, 0.07–0.19) and FF (range for the entire collection, 28.78–89.42), especially for the MIN and FIA genotypes that resulted in those with significantly higher values for these traits in comparison with the other named varieties (Figures 3F,G). However, it should be noted that within the named varieties, COR landraces showed significantly lower PI and FF values. In fact, the lowest PI value of the entire collection (0.07) was found for COR2 accession. Although a range from 3.4 (SPA1) to 7.5 (PER) was found across the entire collection for the °B value, no significant differences were observed for this trait among the named varieties subjected to ANOVA (Figure 3H).

### Correlation Among Traits

**FIGURE 4:**
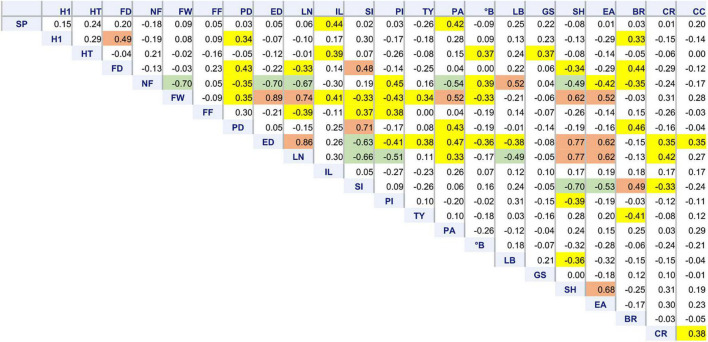
Correlation matrix of 22 morphological traits. Pearson’s coefficients calculated among 64 tomato accessions for variables showing at least one significant correlation, including stigma position (SP), plant height at the first node (H1), total plant height (HT), flowering date (FD), number of fruits (NF), fruit weight (FW), fruit firmness (FF), polar (PD) and equatorial (ED) diameter, locule number (LN), internode length (IL), fruit shape index (PI), pericarp index (PI), total yield (TY), puffiness appearance (PA), total soluble solids (°B), leaf border (LB), green shoulder (GS), fruit shoulder shape (SH), easiness to detach (EA), blossom end rot (BR), radial (RC), and concentric (CC) cracking. Coefficients significant for *p* ≤ 0.01 are highlighted in yellow. Significance for *p* ≤ 0.0001 is highlighted in green or brown if negative or positive, respectively.

Correlation among traits revealed the relationships expected for dimensional and structural fruit traits. Plants having big, flattened fruits (low SI, high LN, and ED) showed a lower NF, PI,°B, and FF and a higher susceptibility to cracking (Figure 4). Such varieties also tended to have a later flowering and higher PA, SH, and EA. They, however, showed the highest yield, being TY more dependent on FW than on NF.

Among floral traits, SP was positively correlated with IL and PA. Blossom end rot had higher incidence on accessions with higher PD and SI (elongate fruit shape); BR was negatively correlated with TY (Figure 4).

### G × E Interaction, Heritability, and Regression Between Locations

A total of six quantitative fruit traits were measured in parallel in a second cultivation site (Alvito) with different pedoclimatic conditions. The G × E interaction estimated by ANOVA was highly significant for all traits (Table 1).

**TABLE 1:** Results of the two-way analysis of variance based on the main factors Genotype (G) and Environment (E) and broad-sense heritability (h^2^_B_) calculated for the quantitative variables fruit weight (FW), polar diameter (PD), equatorial diameter (ED), shape index (SI), number of locules (LN), and pericarp thickness index (PI) measured in two cultivation environments (Alvito and Viterbo).

Trait	Source of variation						h^2^_B_ estimated at:	
		
	G		E		G × E		Alvito	Viterbo
					
	*F*	*P*	*F*	*P*	*F*	*P*		
FW	42.8	***	47.4	***	3.27	***	0.77	0.72
PD	53.2	***	88.0	***	4.88	***	0.86	0.70
ED	65.7	***	63.4	***	2.49	***	0.82	0.84
SI	86.1	***	2.8	ns	1.99	***	0.85	0.87
LN	56.7	***	30.7	***	2.00	***	0.85	0.82
PI	14.1	***	315.1	***	3.68	***	0.61	0.58

***Indicates factors or interaction significant for p ≤ 0.001; ns, non-significant.

One-way ANOVA allowed to estimate h^2^_B_ in both locations; the highest values, ranging from 0.82 to 0.87, were found for fruit structural traits such as SI and LN. The lowest heritability values were found for PI in both locations, indicating a stronger environmental effect on this trait (Table 1).

**FIGURE 5:**
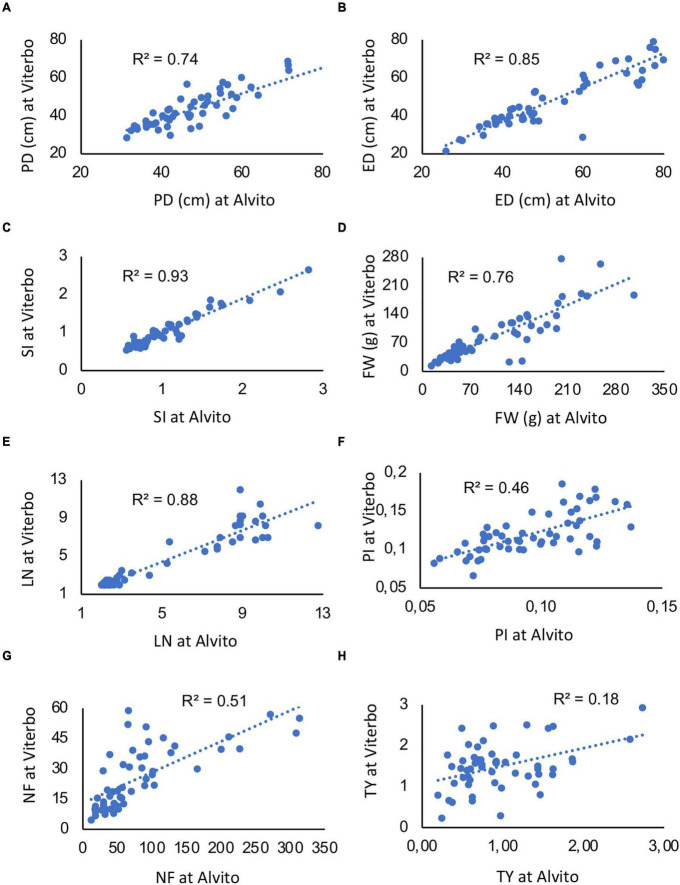
Linear regressions among quantitative traits measured on the 64 studied accessions in two cultivation environments, Viterbo and Alvito. **(A)** Polar diameter (PD), **(B)** equatorial diameter (ED), **(C)** fruit shape index (SI), **(D)** fruit weight (FW), **(E)** locule number (LN), **(F)** pericarp index (PI), **(G)** fruit number (NF), and **(H)** total yield (TY). The determination coefficient (*R*^2^) is reported within each panel; all regressions are significant for *p* ≤ 0.001.

Notwithstanding the significant interaction, linear regressions of the same trait measured in two locations were highly significant for five traits (Figures 5A–E), with an *R*^2^ value ranging from 0.74 to 0.93. Thus, G × E interaction was due to few single genotypes. However, when PI, NF, and TY were considered, the *R*^2^ value was lower (0.46, 0.51, and 0.18, respectively) though still significant (Figures 5F–H).

### Distinctiveness Based on Phenotypic Descriptors

To establish distinctiveness among named landraces, and attribution or exclusion of the unnamed ones, the flat-type and non-flat-type groups were considered separately, and multivariate analysis was performed within each group. The PCA carried out on the flat-type group highlighted that the first two components explained 37.2% of the total variance, with 25.6 and 11.6%, respectively (Supplementary Figure 2A). FW and SI were the main factors discriminating the flat-type accessions accounting for 13.3 and 17.6% of the total variation of the PC1 and PC2, respectively (Supplementary Figure 2B).

**FIGURE 6:**
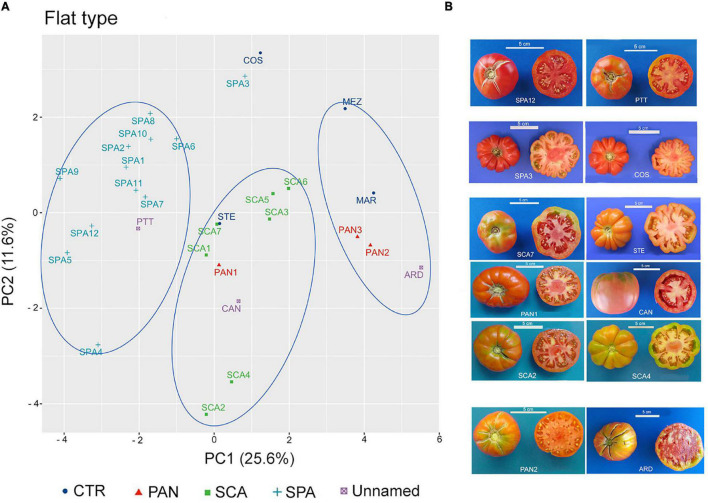
Phenotypic variability of the 29 tomato genotypes included in the flat-type group. **(A)** Scatter plot of the first (PC1) and second (PC2) principal component, showing the variation in 15 quantitative and ten qualitative vegetative and reproductive traits in the 29 accessions included in the flat-type group. Accessions are represented by different colored symbols as indicated in the legend; abbreviations are listed in Supplementary Table 1. **(B)** Pictures of whole and cross-sectioned fruits of representative accessions included in each of the three clusters identified by the PCA. Scale bar is 5 cm.

In the graphical representation of the PCA, three main clusters could be identified (Figure 6A). The first showed negative PC1 values and included the unnamed accession PTT and all the accessions belonging to the SPA landrace except for SPA3, which was localized close to the control genotype COS. The accessions included in this cluster were characterized for having potato leaf (Supplementary Table 5) and for producing fruits with lower FW and SI. SPA3 differed at phenotypic level from the other SPA accessions for having higher value for RI and PA traits and lacking the potato leaf trait; as such, it was like the COS genotype (Supplementary Table 5 and Figure 6B). The second cluster, localized around the middle of PC1, comprised all the SCA accessions as well as the control genotype STE and the unnamed accession CAN. The named landrace PAN1 was also localized within this group. The third cluster was identified by PC1 values > 2; the named landraces PAN2 and PAN3 were found together with the control cultivar MAR and the unnamed accession ARD. According to the descriptor contribution for the two PC, accessions belonging to this cluster were characterized for producing fruits with the highest FW among the accessions included in the flat-type group (Supplementary Table 6).

In the non-flat-type group, the first two components explained about 40% of the total variance, with 23.5 and 16.6%, respectively (Supplementary Figure 3A). NF, PD, PA, and ED were the main descriptors discriminating the analyzed accessions; the first three accounted for 13, 12, and 10% of the total variation of PC1, respectively, whereas ED accounted for 16% of the total variation of PC2 (Supplementary Figure 3B).

**FIGURE 7:**
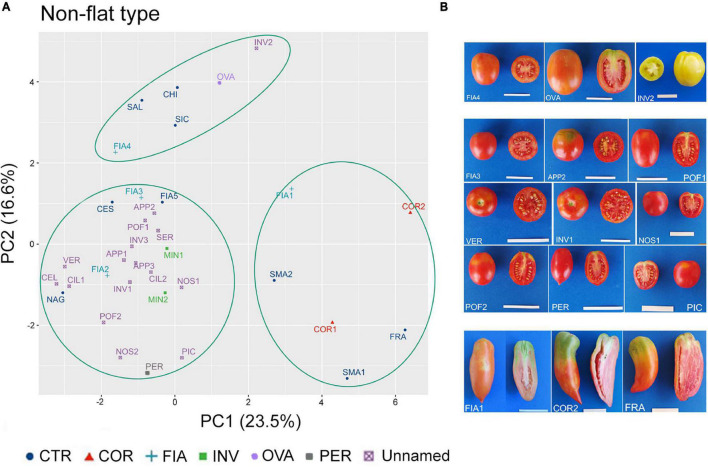
Phenotypic variability of the 35 tomato genotypes included in the non-flat-type group. **(A)** Scatter plot of the first (PC1) and second (PC2) principal components showing the variation of 15 quantitative and ten qualitative vegetative and reproductive traits in the 35 accessions included in the non-flat-type group. Accessions are represented by different colored symbols as indicated in the legend; abbreviations are listed in Supplementary Table 1. **(B)** Pictures of whole and cross section fruit of representative accessions included in each of the three clusters identified by the PCA. Scale bar is 5 cm.

Although the dot plot chart of the PCA highlighted a broad diffusion of the accessions, three main clusters could be identified (Figure 7A). At the top left dial, a first cluster included the named landraces FIA4 and OVA, the unnamed accession INV2, and the control cultivars CHI and SAL. According to their location in the chart, these accessions were characterized for producing fruits with a higher ED (rectangular fruit shape) in comparison with the other non-flat-type accessions (Figure 7B). The second cluster was placed at the bottom left dial and contained most of the non-flat-type accessions, including the FIA, MIN, and PER named landraces, 15 of the unnamed accessions, along with the control genotypes CES, FIA5, and NAG. These accessions differentiated for the low FW and round fruit shape (Supplementary Tables 5, 6) and were characterized for higher NF and lower PD and PA. The third group was localized at the bottom right of the chart and did not encompass any unnamed accessions; in this cluster, only accessions of the named landraces COR and FIA (FIA1) and the control genotypes SMA1, SMA2, and FRA were observed (Figure 7A), all characterized by elongate fruits (Figure 7B and Supplementary Table 5).

### Distinctiveness Based on Molecular Data

The 64 accessions of the studied collection were screened by 7,720 SNPs of the SolCAP array panel. After filtering, a total of 4,679 SNPs and 59 accessions were retained; with this matrix, the analysis was performed. The rejected accessions included the named landrace SCA2, the unnamed accessions INV3 and POF2, and the two control genotypes CES and NAG. Overall, 2,154 out of the 4,679 SNPs showed a MAF < 0.05; hence, more than half of the analyzed sites exhibited a high major allele frequency.

Ho ranged from 0 to 0.17 with a mean of 0.009; the highest value was found in APP2 (Supplementary Table 7). When screened for alleles at sites containing the resistance genes mostly used in breeding (*Pto*, *Cf-2*, *Mi-2*, *Ve1*, *Tm-2^2^*, *Sw-5*, *I*, and *I2*), 11 accessions showed at least one introgression at one locus (Supplementary Table 7). The most reported introgression was for locus *I*, whereas the accession with the highest number of introgressions was CIL1.

**FIGURE 8:**
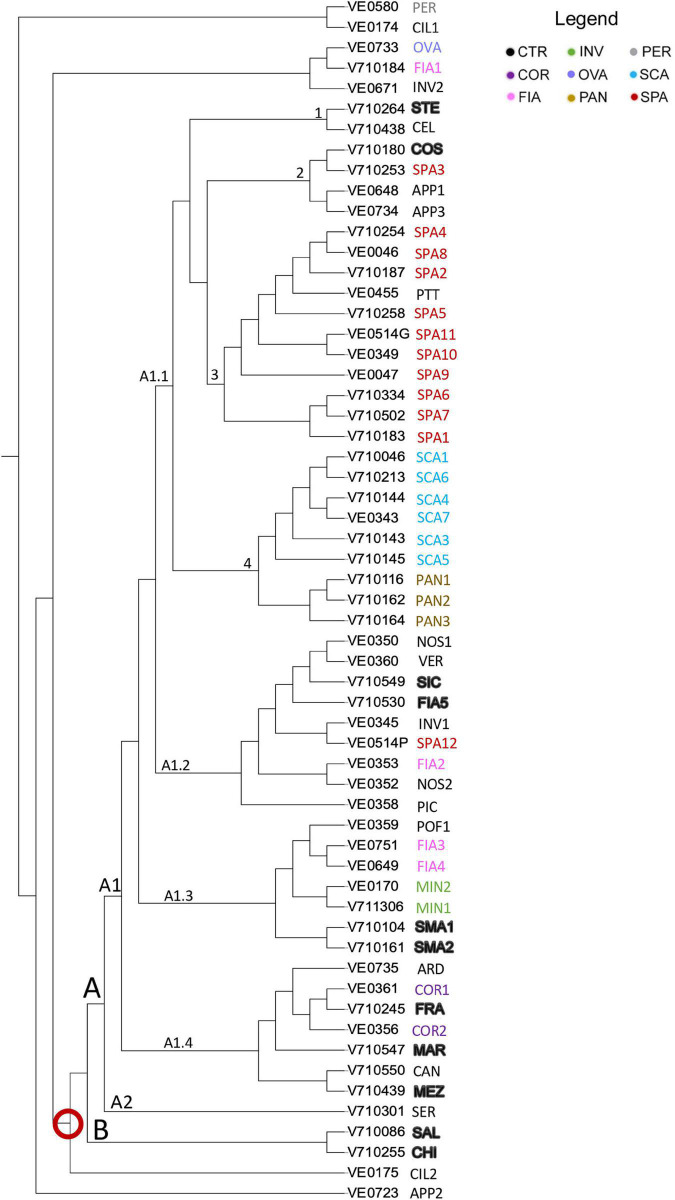
Dendrogram of genetic relationships of 59 tomato accessions based on SNP. Neighbor-joining tree analysis generated by TASSEL; the different named accessions group and controls are indicated in different colors as in the legend; codes and abbreviations are listed in Supplementary Table 1. Red ring indicated the main group of the entire collection.

The results of the neighbor-joining tree analysis showed that 53 out of 59 accessions of the collection were placed together in a single large group indicated by the red ring on the neighbor-joining tree (Figure 8). The remaining seven (i.e., APP2, CIL1, CIL2, FIA1, OVA, INV2, and PER) localized separately, and among these, the named landrace OVA grouped together with FIA1 and with the unnamed accession INV2. Such accessions were also listed among those with the highest Ho and with the introgressions at the *Ve1*, *I*, and *I-2* loci.

The large group of the dendrogram immediately differentiated into two groups, named A and B; the latter only including the two controls SAL and CHI. Group A comprised two clusters, A1 and A2. A2 included only the unnamed accession SER. A1 branched out into 4 arms; A1.1 grouped all the flat-type accessions distributed into 4 subclusters (Figure 8). Subcluster 1 encompassed the control genotype STE, together with the unnamed accession CEL, included in the non-flat-type group. Subcluster 2 contained the accession SPA3, the control COS, and the two unnamed varieties APP1 and APP3 not included in the flat-type group. Subcluster 3 contained the unnamed accession PTT and all the SPA accessions except for SPA12, placed in branch A1.2 and SPA3. Subcluster 4 included all the SCA accessions and, separately, the named landrace PAN. Branches A1.2, A1.3, and A1.4 encompassed the non-flat-type accessions. Most of the unnamed ones (i.e., NOS1, NOS2, PIC, INV1, and VER) were placed in branch A1.2, along with the control genotypes FIA5 and *SIC*. The named landraces MIN1, MIN2, FIA3, and FIA4 and the control genotypes SMA1 and SMA2 were found in branch A1.3, together with the unnamed accession POF1. A1.4 comprised the named landrace COR, the control genotypes FRA, MEZ, and MAR, and ARD and CAN, two accessions included in the flat-type group according to the phenotypic analysis (Figure 8).

**FIGURE 9:**
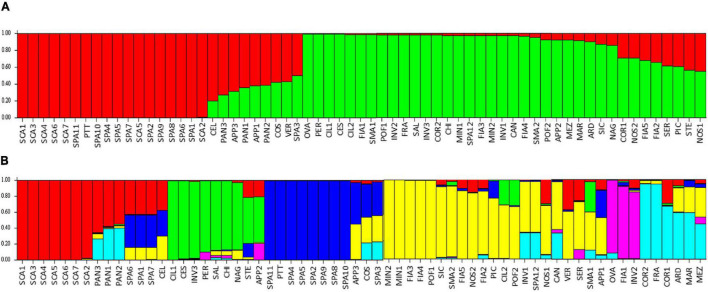
Estimated population structure: each individual is represented by a vertical bar, which is partitioned into colored segments that represent the individual estimated levels of the two **(A)** or six **(B)** clusters. Accessions’ abbreviations are listed in Supplementary Table 1.

The model-based STRUCTURE algorithm was run to investigate the genetic structure of the collection with a subset of 384 unlinked markers. The Δk evaluation indicated support for *k* = 2 and, with a minor strength *k* = 6. When the population was classified according to two clusters, it separated the flat type named varieties SCA, SPA, with PTT, from the non-flat types (Figure 9A). A few flat-type accessions showed an admixed genotype, such as PAN, COS, and STE. The *k* = 6 model better discriminated the varieties (Figure 9B); SCA accessions belonged to a first group (red cluster), PAN was admixed between the SCA group and the group characterized by elongated fruits (COR and FRA, cyan cluster), whereas accessions showing introgressions of resistance genes were grouped separately (green and purple clusters). The SPA accessions formed a separate group (blue cluster), although three of them showed admixture with the SCA cluster and SPA3 confirmed its similarity with COS (Figure 9B).

When the phylogenetic analysis was run adding 40 cultivars and landraces of different origins from a previous study (Sacco et al., 2015; Supplementary Table 3), additional accessions of PAN (K_PAN1, K_PAN2) and SCA (K_SCA1, K_SCA2) mapped consistently with those of our collection (Supplementary Figure 4). As for other named landraces, OVA showed high similarity with two processing cultivars (K_RIO, K_RIM), PER with indeterminate cultivars (K_KIR, K_CAS), COR with an elongate landrace from Abruzzo (K_ALL), MIN and FIA with the landrace Corbarino (K_COR), and other small-fruited accessions from the Campania region (Supplementary Figure 4).

## Discussion

The presented analysis offered a comprehensive description of the genetic variability of the tomato germplasm in a specific region in Central Italy. A wide diversity between flattened and non-flattened-type groups for vegetative and fruit traits was detected in qualitative and quantitative descriptors, confirming the wealth of Italian traditional tomato germplasm (Baldina et al., 2016; Raggi et al., 2022).

In addition to obvious fruit structural traits, the flat group shared common features, such as an entire or undulate leaf border, a prevalently uniparous or forked inflorescence, a light green shoulder, and a strongly depressed fruit shoulder shape, which makes fruit detachment difficult. The incidence of radial cracking was also higher in flat tomatoes. In contrast, non-flat types showed a prevalently serrated leaf border, fishbone inflorescence, medium intense green shoulder, easy fruit detachment, and lower occurrence of radial cracking. Whereas all flat accessions presented the green shoulder, among the non-flat, there were six purposed landraces lacking it (“*uniform*” phenotype), in addition to the processing cultivars CHI and SAL. This may indicate a possible origin of some non-flat accessions from modern, genetically improved material (Vogan, 2012).

Relationships among traits evidenced general defects of the two groups. The flat types were characterized by low values of fruit number, soluble solid content, and pericarp index, resulting in a lower fruit firmness. Within non-flat tomatoes, those having elongate fruits showed higher susceptibility to blossom-end rot; this also negatively affected the total yield.

Conversely, the data offered useful information about the traits that could be useful for traditional germplasm in breeding. The SCA accessions were the firmest among flat-fruited types and in addition presented a constantly inserted stigma, a non-common phenotype among big tomatoes. Regarding fruit quality, the MIN and FIA types showed high soluble solid content. PAN and SCA reached high total yield, comparable to the controls CHI, CAN, and COS, although the reliability of this finding should be assessed on a higher number of plants and with the replicates in different growing seasons.

When the collection was observed in two different environments, a strong G × E interaction was detected for fruit traits; this was, however, due to the behavior of few single genotypes, as already reported (Mazzucato et al., 2008). Thus, the genetic control of these traits is strongly genetically controlled, as also shown by heritability estimation. Productive traits instead showed lower correlation between the two environments. The adoption, and comparison, of two different locations partially make up for the limitation of a single season of data detection. It is, however, to be considered that the two environments belonged to the same region; the phenotypic plasticity of the studied genotypes when grown in very different environments remains to be ascertained.

Because often germplasm collection includes accessions that are not assigned to renown landraces that may represent non-autochthonous or non-traditional genotypes (Gibson, 2009; Cortés-Olmos et al., 2015), a focus of the present work was to assess the distinctiveness among named landraces and to assign (or exclude) an identity to the accessions of uncertain classification. The dual analysis confirmed the congruity of the named flattened-ribbed landraces. Phenotypically, PAN accessions were distinguished by tall plants producing big fruits with higher fruit shape index; the SPA landrace was characterized by smaller plants, with early flowering and small fruits and the “potato leaf” trait; SCA accessions showed intermediate values for internode length and fruit weight, a generally inserted stigma, higher susceptibility to blossom-end rot and the typical occurrence of puffiness. The molecular analysis assisted the attribution or exclusion of uncertain accessions; ARD, which was phenotypically similar to PAN, was instead differently classified after molecular analyses, whereas PTT was classified as SPA by all the analyses. In contrast, SPA3 was not included in the SPA phenotypic and molecular cluster but mapped always close to the control COS, indicating a possible introduction of a “Costoluto fiorentino” type. In addition, SPA12, although being phenotypically like the SPA ideotype, showed a different classification at the molecular level, probably due to introgressions and successive recovery of the potato leaf phenotype. Although falling among the SCA accessions for the phenotype, the unnamed accession CAN could not be ascribed to the SCA landrace for its different genetic backgrounds, being closer to the control landrace MEZ from the neighbor Abruzzo region.

Among the named non-flat types, the phenotypic analysis identified a group with elongate fruits; this included the COR accessions, showing both morphological and molecular similarity to FRA, a landrace from the neighbor region of Umbria, as well as to K_ALL from Abruzzo. These tomatoes are probably the derivatives of the French variety Cornue des Andes, although previous research failed to show a similarity between FRA and the French heirloom (Castellana et al., 2020). This evidence indicates that horn-shaped tomatoes do not represent a landrace native of a specific Italian region.

Other non-flat named varieties were supported by the analyses, such as MIN, which also showed similarity to the FIA3 and FIA4 accessions. Although being genetically similar, the accessions of the FIA and MIN landraces could be distinguished, as FIA had a lower internode length, fruit firmness, and shape index. When compared in a wider scenario, FIA and MIN showed similarity to small-fruited accessions from the neighbor region of Campania, primarily to Corbarino. From both analyses, it was clear that FIA1 was not a true FIA type because of morphological and molecular diversities. FIA1, together with other six purposed landraces, showed several introgressions at sites corresponding to common genes for resistance used in modern breeding (Foolad, 2007), in parallel with a relatively high level of heterozygosity. Such genetic constitution suggested that these accessions were the derivatives of modern materials, after segregation from accidental crosses or commercial F_1_ hybrids. Being tomato a strict self-pollinating species, very low levels of Ho are usually found in true breeding varieties and landraces (Mazzucato et al., 2008; Aflitos et al., 2014; Sacco et al., 2015); thus, an increased frequency of heterozygotes indicates intentional or accidental outcrossing and/or introgressions from wild relatives. Some of these genotypes, such as INV2, PER, and OVA, also showed a morphological signature of modern breeding such as the lack of green shoulder. INV2 had a delayed ripening phenotype, likely being a segregation of a recessive allele used in a hybrid combination. Consistently, OVA and PER clustered with improved cultivars, both vintage (K_RIO, K_RIM, K_SAN) and modern (K_KIR, K_CAS).

Hence, the PER accession included in our collection could not be claimed as representative of a traditional variety. In contrast, finding the OVA landrace among those subjected to breeding was not surprising, as it is known that this tomato represents the rediscovery of ancient selections made in the province of Rieti^7^ (last accessed on 20 April 2022).

Of the remaining unnamed varieties, two accessions shared the geographical appellative “Pomodorella di Pofi,” of which POF1 was very similar to two FIA accessions and the other was omitted from the molecular analysis because of very high heterozygosity. If POF1 represents the true type of this landrace, it should be considered very close to FIA, as also the phenotypic analysis showed.

The accession VER, having a geographical appellative, was molecularly close to NOS1, an unnamed accession collected in the same geographical area; they may thus share a common genetic background and represent a candidate new local variety.

Overall, the data indicate that the phenotypic and genotypic approaches are complementary and necessary for a better distinction of landraces and for the attribution or exclusion of unnamed accessions. Although the genotypic analysis is based on a high number of markers, it should be feasible to select the minimum set of variations sufficient to draw the membership to a named landrace.

## Conclusion

The reported analysis highlighted the high variability of tomato landraces which have been found in a specific region of Central Italy. Phenotypic and genetic information provided in this study will be very useful to preserve and valorize those traditional tomatoes, including (i) to supply efficient descriptors to direct the seed producer, the farmer, and the consumer; (ii) to juridically protect the landraces; (iii) to assign (or exclude) new collections to named varieties; and (iv) to indicate to the breeder the genetic-molecular boundaries to drive the eventual improvement of local varieties.

## Data Availability Statement

The variant data for this study have been deposited in the European Variation Archive (EVA) at EMBL-EBI under accession number PRJEB54712 (https://www.ebi.ac.uk/eva/?eva-study=PRJEB54712).

## Author Contributions

AM and MEP: conceptualization. BF, MEP, FS, RR, and PT: formal analysis. BF, FS, and RR: data curation. AM and BF: writing and original draft preparation. AM and PT: funding acquisition. All authors read and agreed to the published version of the manuscript.

## Conflict of Interest

The authors declare that the research was conducted in the absence of any commercial or financial relationships that could be construed as a potential conflict of interest.

## Publisher’s Note

All claims expressed in this article are solely those of the authors and do not necessarily represent those of their affiliated organizations, or those of the publisher, the editors and the reviewers. Any product that may be evaluated in this article, or claim that may be made by its manufacturer, is not guaranteed or endorsed by the publisher.

## Footnotes

1
https://www.sian.it/mivmPubb/listeVarieta.do

2
https://www.arsial.it/biodiversita/registro-volontario-regionale/

3
https://www.politicheagricole.it/flex/cm/pages/ServeBLOB.php/L/IT/IDPagina/16681

4
https://www.slowfood.com/presidia

5
https://www.vhlgenetics.com/en-gb/

6
https://github.com/kassambara/factoextra/issues

7
https://www.visitrieti.com/2020/03/18/tomato-eel/
