# Supplementary material for: Phenotypic and Genotypic Diversity of the Tomato Germplasm From the Lazio Region in Central Italy, With a Focus on Landrace Distinctiveness
Source: Front Plant Sci. 2022 Jul 22;13:931233. doi: 10.3389/fpls.2022.931233 (PMC9355589; doi:10.3389/fpls.2022.931233)
Supplement: Supplementary file 2 [file Data_Sheet_2.docx]

Supplementary Material

# Supplementary Figures


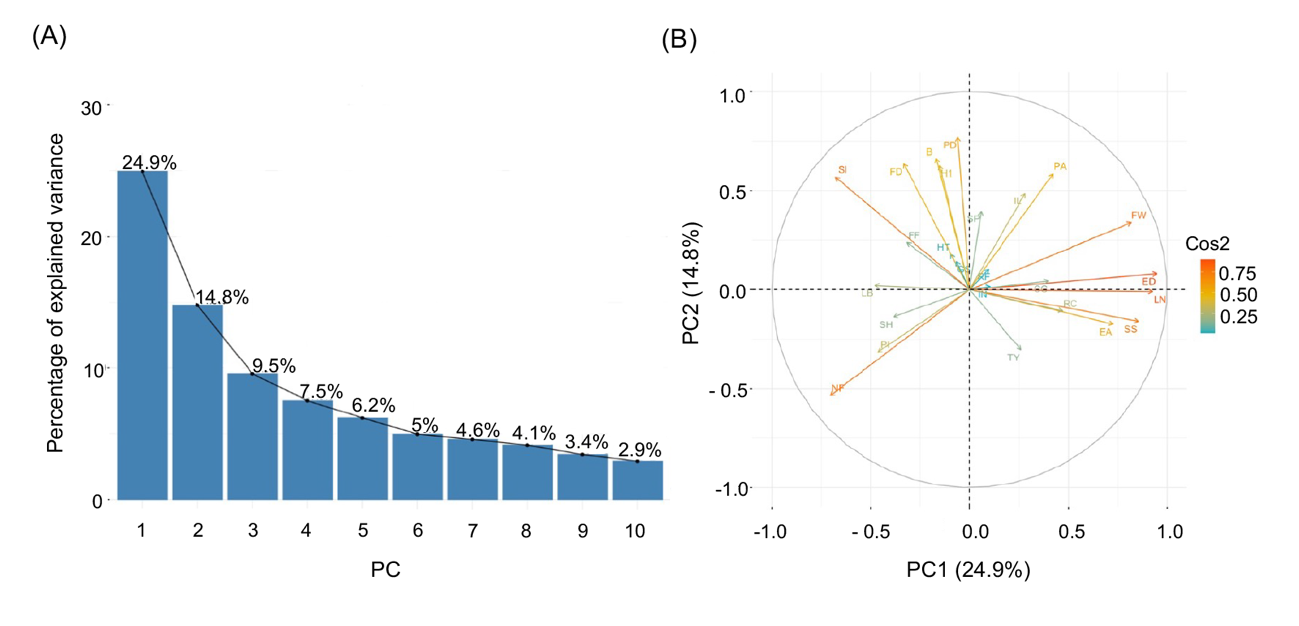


- **Supplementary Figure 1.** **Principal component analysis carried out on the whole population.** **(A)** Percentage of variation explained by each principal component (PC); the first six component explained 68.0% of the variation, the first (PC1) and second (PC2) component explained 24.9% and 14.8% of the total variance. **(B)** Contribution of qualitative and quantitative descriptors to the first two principal components. The colorimetric scale indicates the different variable contribution value, according to the gradient color. Red, variable contribution > 0.75; yellow, variable contribution 0.25–0.75; cyan, variable contribution > 0.25. Descriptors’ acronyms are listed in Supplementary Table 2.


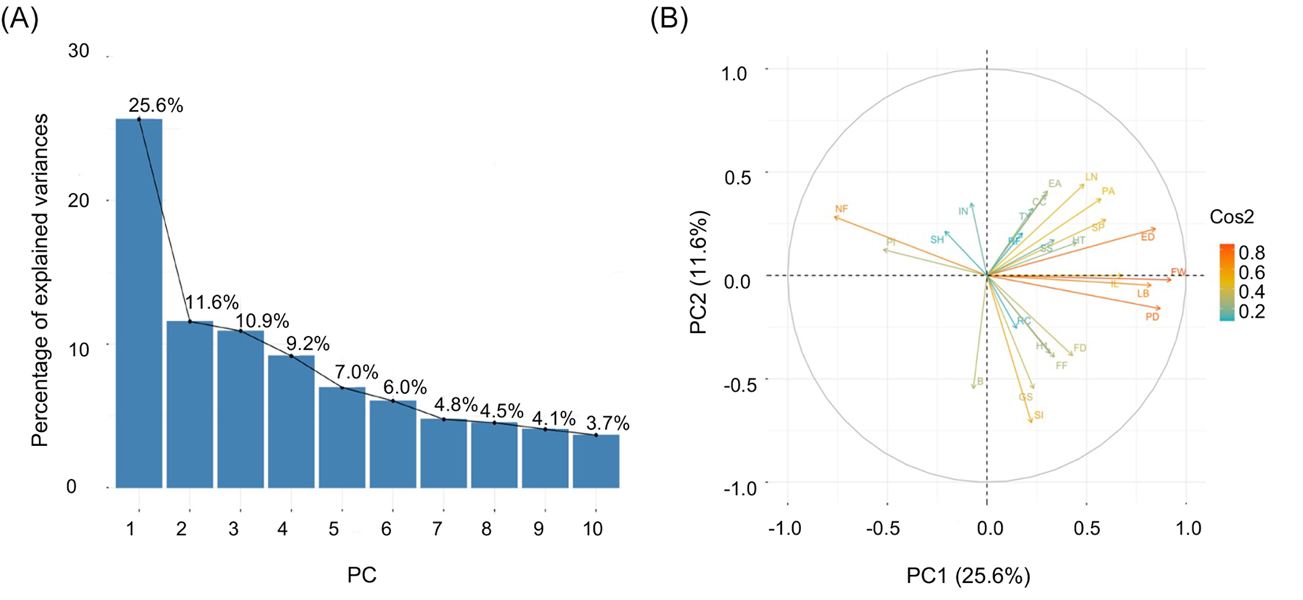


- **Supplementary Figure 2.** **Principal component analysis carried out on the 29 accessions with flat fruits.** **(A)** Percentage of variation explained by each principal component (PC). PC1 and PC2 explained 25.6% and 11.6% of the total variance, respectively. **(B)** Contribution of qualitative and quantitative descriptors for the first two principal components. The color scale indicates the different variable contribution value, according to the gradient color. Red, variable contribution value ≥ 0.8; yellow, variable contribution value 0.2–0.6; cyan, variable contribution value < 0.2. Descriptors’ acronyms are listed in Supplementary Table 2.


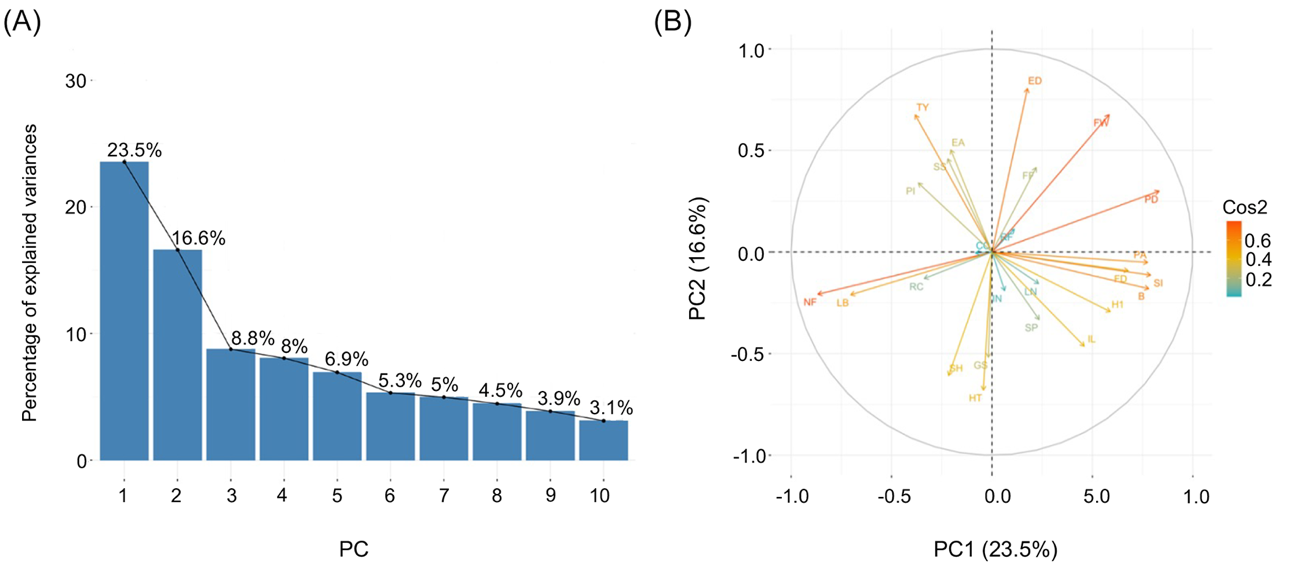


- **Supplementary Figure 3. Principal component analysis carried out on the 35 accessions with non-flat fruits. (A)** Percentage of variation explained by each principal component (PC). The first six principal component (PC) explained 69.2% of the variation. PC1 and PC2 explained 23.5% and 16.6% of the total variance, respectively. **(B)** Contribution of qualitative and quantitative descriptors for the first two principal components. The color scale indicates the different variable contribution value, according to the gradient color. Red, variable contribution value > 0.75; yellow, variable contribution value 0.2–0.6; cyan, variable contribution value < 0.2. Descriptors’ acronyms are listed in Supplementary Table 2.


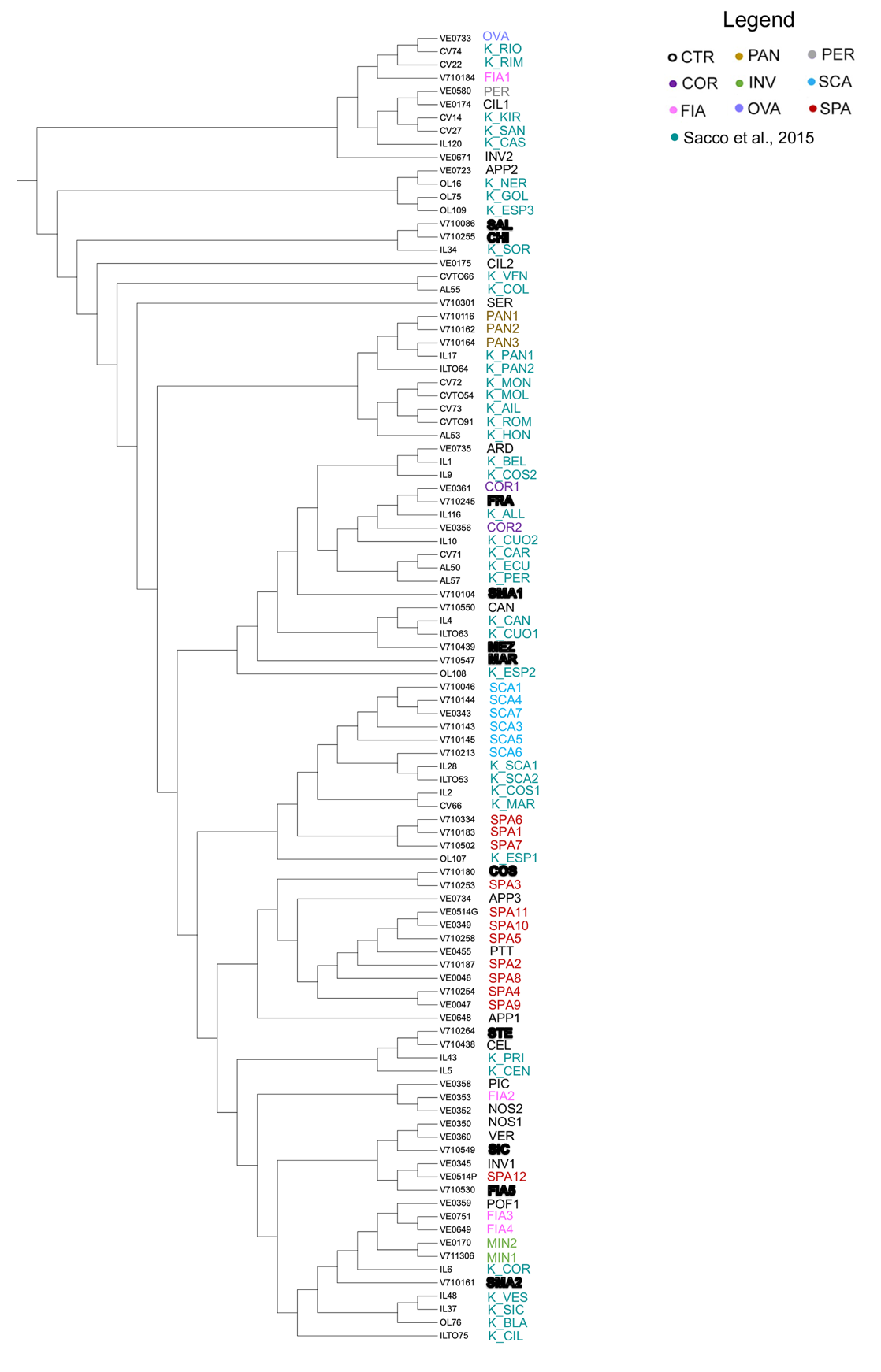


- **Supplementary Figure S4**: **Dendrogram of genetic relationships of tomato accessions from our collection integrated with 40 genotypes from Sacco et al., 2015, based on SNP polymorphisms**. Neighbor-Joining tree analysis generated by TASSEL; the different named accessions group and controls are indicated in different colors as in the legend; codes and abbreviations are listed in Supplementary Tables 1 and 3
